# CREATING INCLUSIVE COMMUNITIES FOR LGBTQ RESIDENTS AND STAFF IN FAITH-BASED ASSISTED LIVING COMMUNITIES

**DOI:** 10.1093/geroni/igae098.1615

**Published:** 2024-12-31

**Authors:** Carey Candrian

**Affiliations:** University of Colorado Anschutz Medical Campus, Denver, Colorado, United States

## Abstract

Seventy-five percent of older LGBTQ adults report having to hide their identity (and therefore, their true selves) in order to avoid discrimination in long term care settings. The stress of hiding takes a serious toll on the lives of LGBTQ people: higher rates of anxiety and depression, substance abuse, certain cancers, cardiovascular disease and suicide. Knowing first-hand the pain of not belonging, residents at a faith-based assisted living community in the Rocky Mountain Region have been working to create a more welcoming community for LGBTQ residents since 2017. Their LGBTQ + Friends group is made of LGBTQ people and allies (residents and team members) who meet monthly to create an inclusive and safe space for all. A few years ago, the group asked, what could help long term care communities become a more welcoming place for LGBTQ residents and staff? Through this community-initiated idea, and after foundation funding, we developed a two-year cultural change initiative for over 800 staff across 6 assisted living communities called, “Caring for all: Opening hearts and minds.” The result is a 25-minute video of voices of residents, staff and leadership talking about the impact of creating inclusive communities where all residents can belong and be embraced. This project was not without enormous challenges and barriers. During this presentation, we will also walk through lessons learned and stories of success for creating inclusive communities for LGBTQ residents and staff in faith-based assisted living communities.

